# Supplementary material for: Superelasticity and cryogenic linear shape memory effects of CaFe2As2
Source: Nat Commun. 2017 Oct 20;8:1083. doi: 10.1038/s41467-017-01275-z (PMC5715139; doi:10.1038/s41467-017-01275-z)
Supplement: Supplementary file 1 — Supplementary Information [file 41467_2017_1275_MOESM1_ESM.pdf]

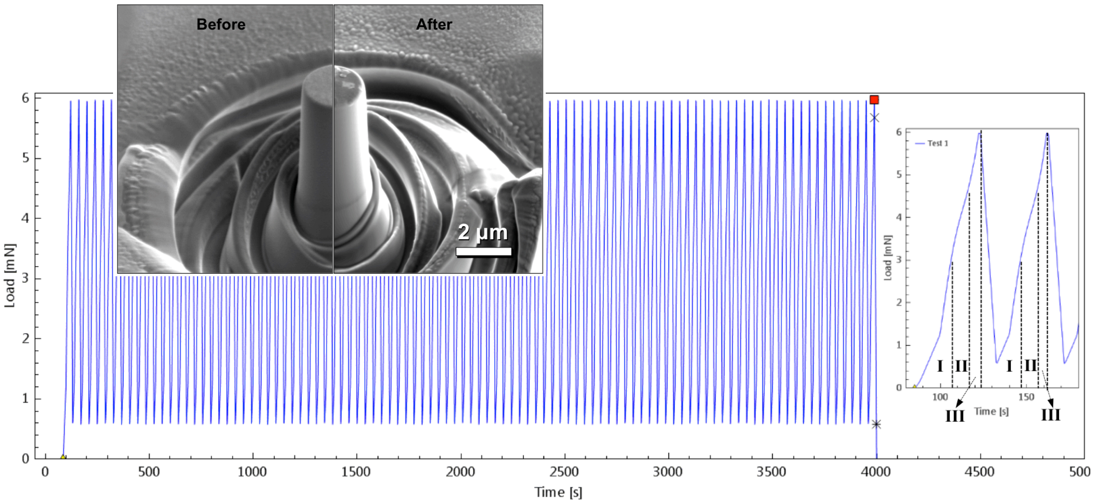

Supplementary Figure 1: 100 cyclic compression test. (a) The time-load curve of 100 cyclic compression test. The non-linear curve of the first two cycles in the inset clearly shows that cT phase transformation occurs every cycle. Inset is $CaFe_2As_2$ micropillar before and after 100 cycles of forward-backward transformation. This image shows that there is no residual plastic deformation after 100 cycles.

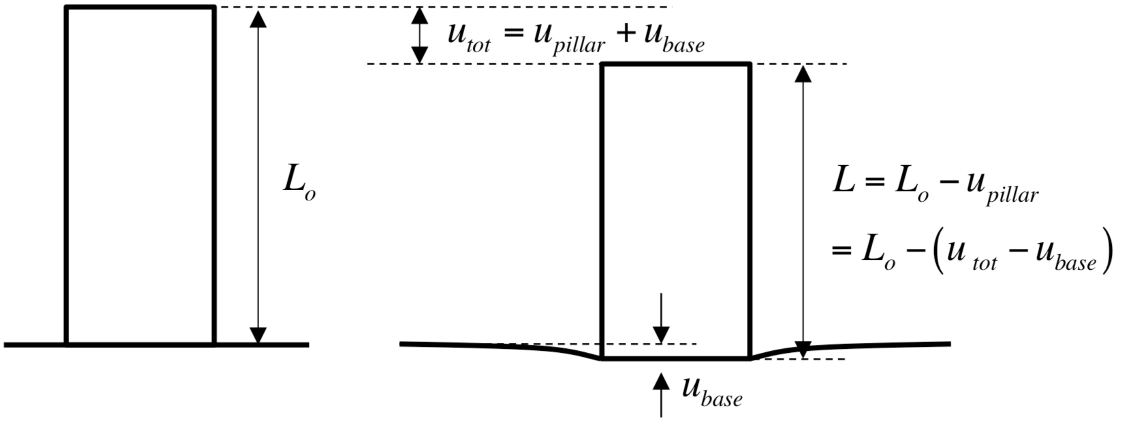

Supplementary Figure 2: Schematic diagram of the deformed micropillar. In order to obtain the precise strain value in the micropillar, it is important to measure the displacement of the base under the micropillar because the total measured displacement (u_{tot}) includes both the displacement of micropillar (u_{pillar}) and the base (u_{base}).

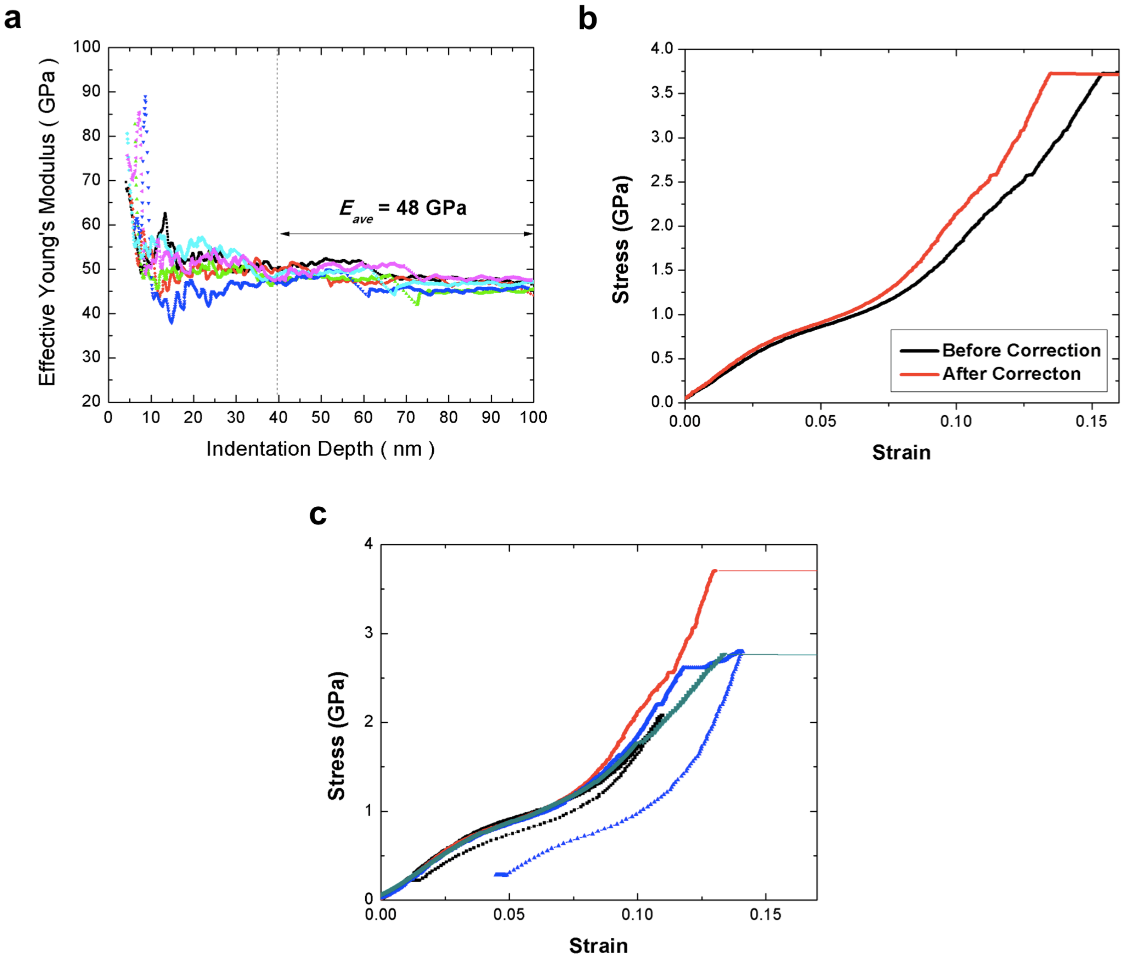

Supplementary Figure 3: Strain correction via Sneddon's flat punch method. (a) The measurement of the effective Young's modulus of [0 0 1] $CaFe_2As_2$, and (b) the correction of stress-strain curve by considering the displacement of the micropillar base (c) Stress-strain curves of four different samples. Note that four stress-strain curves show consistent superelasticity results.

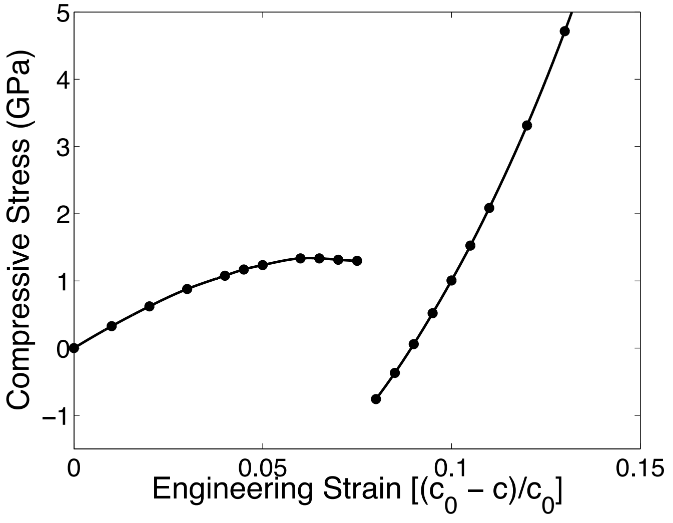

Supplementary Figure 4: A uni-axial stress-strain curve of a single unit cell of $CaFe_2As_2$. At about 8% uniaxial compression, the cell changes to the collapsed tetragonal unit cell with a reduced c-axis length. The reference value for c_0 is taken as the equilibrium value for c in the orthorhombic structure. The stress changes abruptly and the moment on the iron atoms vanishes.

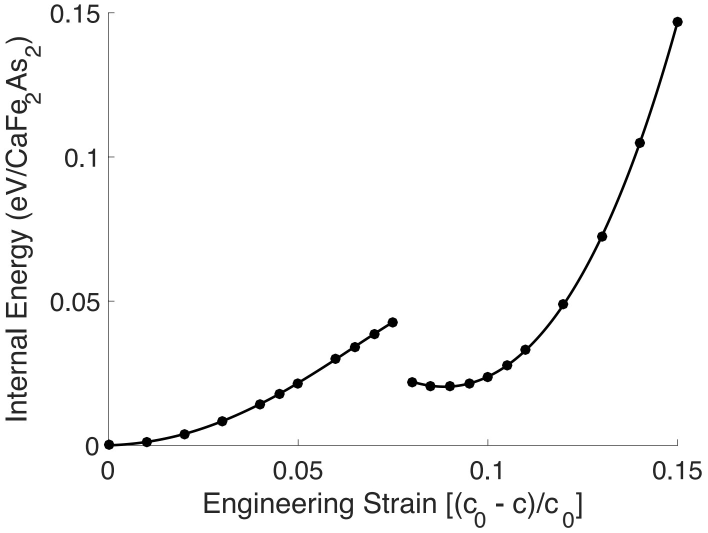

Supplementary Figure 5: A plot of the internal energy of $CaFe_2As_2$ under uniaxial loading. The energy is evaluated at each indicated strain and is plotted per formula unit of $CaFe_2As_2$. Note that the lateral stresses were allowed to relax during uniaxial loading to relieve the lateral stresses.

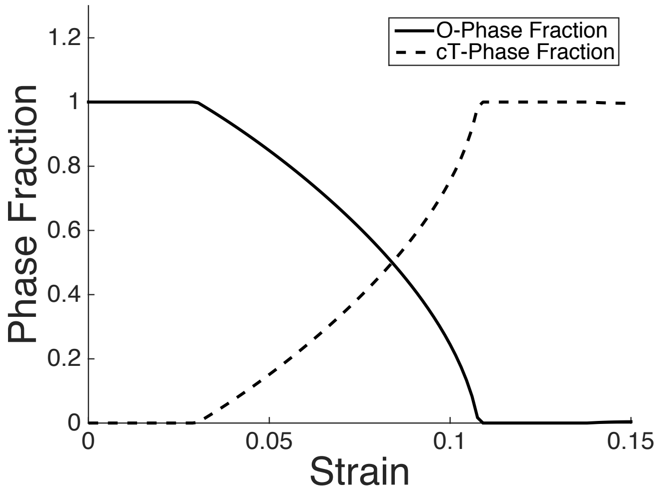

Supplementary Figure 6: A plot of the phase fractions of the two phases as a function of the total strain. The phase fraction evolves during the phase transition and the two phase fractions add to a total of 1.0.

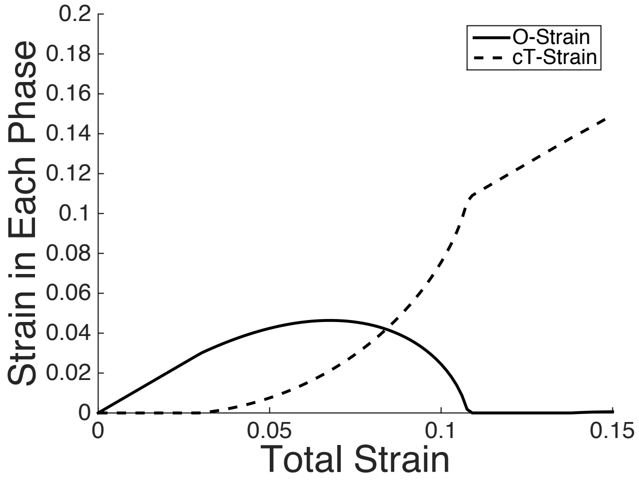

Supplementary Figure 7: A plot of the evolution of the strain in each phase as a function of the total strain. The engineering strain used here is defined relative to the c-axis of the Orthorhombic structure shown in Supplementary Fig. 4 and thus is not the elastic strain in the cT phase but is computationally easier to use. In this case, the strains in the two phases must add to the total strain.

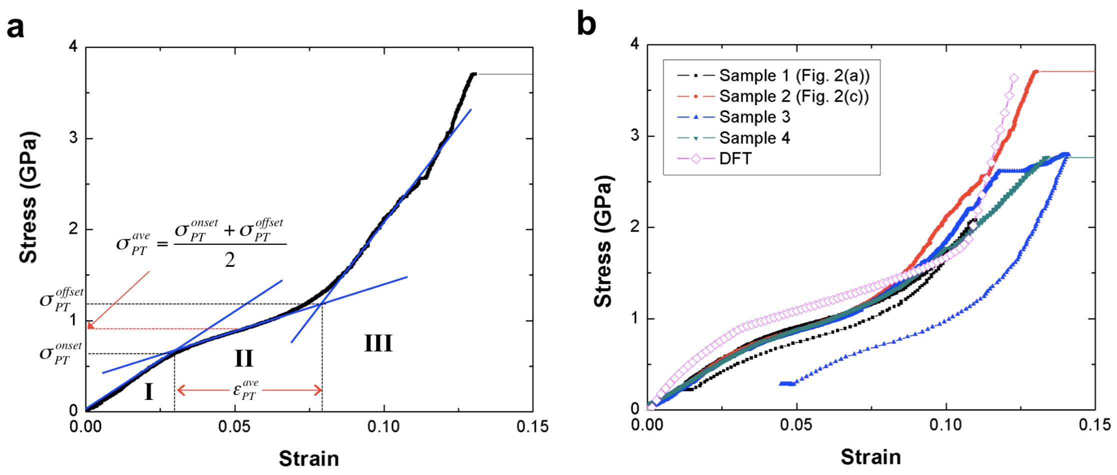

Supplementary Figure 8: Analysis of onset and offset stress of phase transformation. (a) Determination of σ_{PT}^{onset}, σ_{PT}^{offset}, and ε_{PT}^{ave}, (b) four experimental and one computational stress-strain data. Note that Sample 3 underwent the plastic deformation, and did not fully recover to the original shape. However, the elastic portion (stage I, II, III) is full recovered.

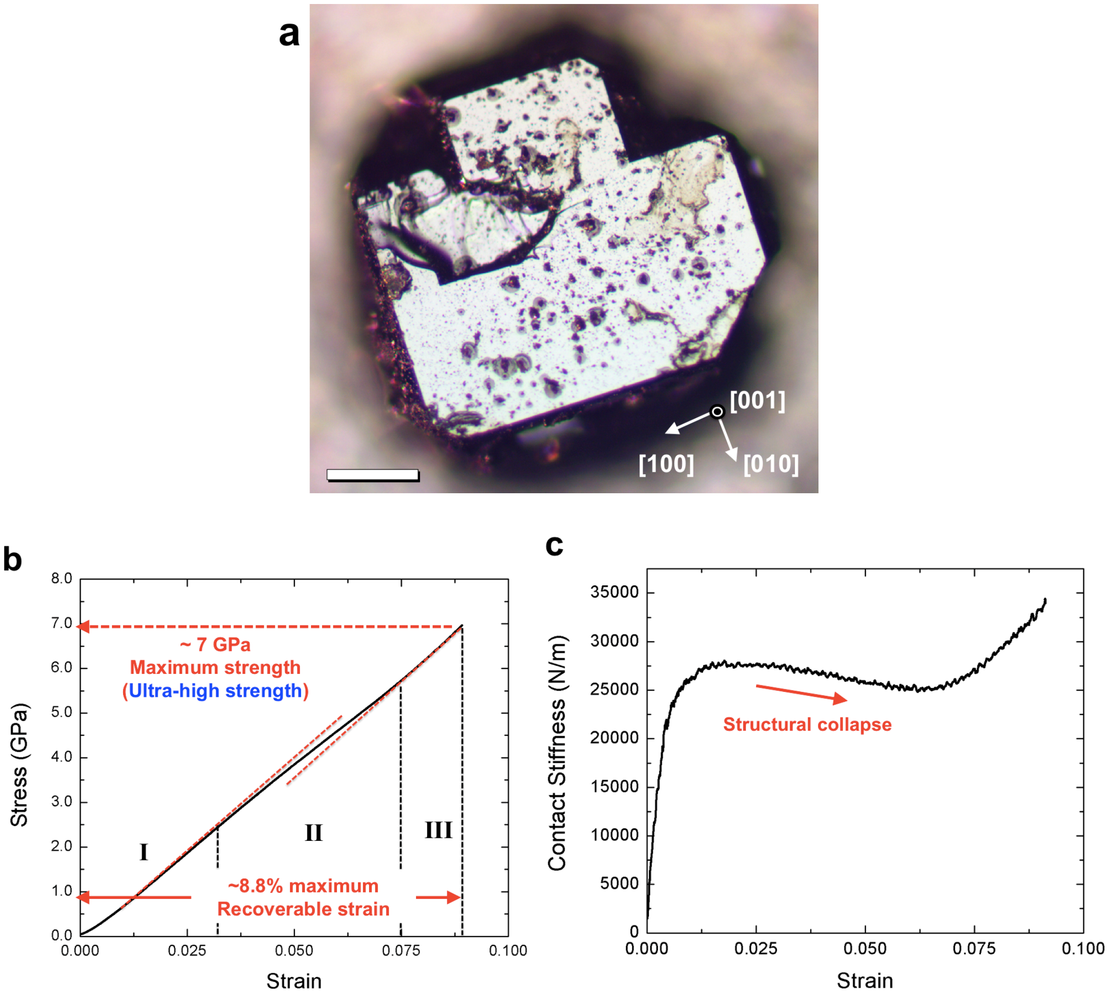

Supplementary Figure 9: Superelastic intermetallic compound, $LaRu_2P_2$. (a) Optical image of single crystalline $LaRu_2P_2$; scale bar, 100 μm (b) Stress-strain curve of [0 0 1] $LaRu_2P_2$. $LaRu_2P_2$ also exhibit the distinct three stages of deformation, which is the characteristic of superelastic and shape memory material (*not published*). (c) Contact stiffness as a function of strain. The decrease in contact stiffness implies that $LaRu_2P_2$ is collapsed during deformation.

	σ_{PT}^{onset} (MPa)	σ_{PT}^{offset} (MPa)	σ_{PT}^{ave} (MPa)	ε_{PT}^{ave}	W_{PT}^{ave} ($\times 10^6$ J/m^3)
Sample 1	752	1321	1036.5	0.055	56.6
Sample 2	700	1286	993	0.052	51.5
Sample 3	673	1269	971	0.0575	55.8
Sample 4	690	1252	971	0.0546	53.0
DFT	852	1711	1281	0.079	101.2

Supplementary Table 1: Calculation of actuation work per-unit-volume (W_{PT}^{ave}). Here, we calculate W_{PT}^{ave} by $\sigma_{PT}^{ave} \times \varepsilon_{PT}^{ave}$, where σ_{PT}^{ave} and ε_{PT}^{ave} are the average transformation stress and strain, respectively.

Supplementary Note 1: 100 cyclic compression test

We performed the 100 cyclic test with 20 nm/sec of displacement rate and 6 mN of maximum load and 0.6 mN of minimum load, and the time-load curve in the inset clearly shows that our micropillar experiences all three stages (I, II and III) for each cycle (Supplementary Fig. 1). So, the forward and backward phase transformation should occur every cycle[3]. SEM images before and after the cyclic test confirms no height change. Thus, $CaFe_2As_2$ does not shows any residual deformation after 100 cycles, which implies the fatigue resistance better than that shape memory ceramic micropillars that typically fails before 5~30 cycles of forward-backward transition. As discussed in the manuscript, cT transition occurs by simple bond adhesion between As layers, which would not introduce a significant stress concentration in the course of phase transformation. Also, we expect a superior fatigue resistance.

Supplementary Note 2: Stress-Strain Calculations

In order to obtain the precise strain value in the micropillar, it is important to measure the displacement of the base under the micropillar because the total measured displacement (u_{tot}) includes both the displacement of micropillar (u_{pillar}) and the base (u_{base}) (Supplementary Fig. 2),

$$u_{pillar} = u_{tot} - u_{base} \quad . \tag{1}$$

The displacement of the base can be calculated by using Sneddon flat punch solution by assuming that cylindrical flat punch of $CaFe_2As_2$ indents the elastic half space of $CaFe_2As_2$[1]. The contact stiffness ($k_{Sneddon}$) from Sneddon solution gives

$$k_{Sneddon} = \frac{2E}{\left(1-\nu^2\right)}\sqrt{\frac{A_{contact}}{\pi}} \quad , \tag{2}$$

where E is the Young's modulus of $CaFe_2As_2$, ν is the Poisson's ratio of $CaFe_2As_2$, and $A_{contact}$ is the contact area between micropillar and the base. The displacement of the base can be given by

$$u_{base} = \frac{P}{k_{Sneddon}} = \frac{P}{\frac{2E}{\left(1-\nu^2\right)}\sqrt{\frac{A_{contact}}{\pi}}} = \frac{P\left(1-\nu^2\right)}{2E}\sqrt{\frac{\pi}{A_{contact}}} \quad , \tag{3}$$

where P is the applied force. Then, the engineering strain can be calculated by

$$\varepsilon_{eng} = \frac{u_{pillar}}{L_o} = \frac{u_{tot} - u_{base}}{L_o} = \frac{1}{L_o}\left[u_{tot} - \frac{P\left(1-\nu^2\right)}{2E}\sqrt{\frac{\pi}{A_{contact}}}\right] \quad , \tag{4}$$

where L_o is the initial height of micropillar.

Here, please note that caution is needed when we use Young's modulus because it is not correct if we use simply the Young's modulus of tetragonal phase only. We must consider the contributions of elastic displacement of the tetragonal phase, elastic displacement of the collapsed tetragonal phase, and the phase transformation displacement to the total displacement of the base. Thus, it is necessary to measure the effective Young's modulus that includes all these contributions under the condition of indentation, and it is possible to obtain it by performing nanoindentation on $CaFe_2As_2$ along c-axis. Poisson's ratio of intermetallic compound ranges typically from 0.2~0.3. Here we assumed $\nu = 0.2$. Note that the contact stiffness of the Sneddon solution is not sensitive to Poisson's ratio, so our Poisson's ratio ($\nu = 0.2$) is a reasonable approximation. With all of this in mind, we were able to obtain the effective Young's modulus by using the Oliver-Pharr method[2]. The nanoindentation data is shown in Supplementary Fig. 3(a), and the average Young's modulus is about 48 GPa. Our DFT simulation shows that Young's modulus of orthorhombic phase, which would be similar with tetragonal phase, is about 33 GPa, and that of collapsed tetragonal phase is about 103 GPa. It makes sense that our result (48 GPa) is in between these values because the tetragonal and collapsed tetragonal phases co-exist in the base during the nanoindentation. Then, the stress-strain curve can be corrected by using Supplementary Equation S4 (Supplementary Fig. 3(b)). All stress-strain curves in this work were corrected in this manner (Supplementary Fig. 3(c)).

Supplementary Note 3: DFT Calculations

Unit Cell Compression

In order to understand the uniaxial deformation of orthorhombic $CaFe_2As_2$, we used DFT to simulate the uniform straining of a single unit cell. It is important to note that at room temperature, the conditions in which the experiments were conducted, the phase transitions observed occur between the paramagnetic tetragonal phase and the non-magnetic collapsed tetragonal phase. All of the DFT calculations, which are done at 0K, involve the transition between the antiferromagnetic orthorhombic phase and the collapsed tetragonal phase. Here, we use the orthorhombic phase as a surrogate for the high temperature paramagnetic tetragonal phase since these two phases have similar lattice constants, with c being the most important, and should have similar bonding with the largest difference being the disordering of the magnetic moments, which cannot be simulated using DFT with current computer resources. The use of the orthorhombic phase as a surrogate for the tetragonal phase will include some error, and thus all the DFT simulation results should be interpreted as an approximation rather than true one-to-one match with experiments.

Our uniaxial deformation studies differ from previous ones in that instead of conducting a simulation where the uniaxial stress was held at fixed value and the lateral stresses were set to zero, these simulations are conducted with mixed boundary conditions. We prescribe a uniaxial strain along the $CaFe_2As_2$ c-axis and the stress is relaxed along the a- and b-axes. This allows us to specifically probe the behavior of $CaFe_2As_2$ as a function of strain and removes some of the issues that can arise with prescribe stress paths during mechanical deformation. However, we note that VASP is

incapable of performing such calculations and a script was written to specifically adjust the lateral dimensions to reduce the lateral stresses to zero. The resulting stress – engineering strain curve is shown in Supplementary Fig. 4.

Composite Stress-Strain Curve

In order to investigate the transition from orthorhombic (O) to collapsed tetragonal (cT) phases in DFT (tetragonal to collapsed tetragonal in experiments) we have attempted to model the response of the $CaFe_2As_2$ pillar assuming that it can be comprised of two phases: O and cT. We assume that at a given macroscopic strain, ε, the system will assume the composition of the O and cT phases that minimizes the free energy under the assumption of mechanical equilibrium. We choose the a free energy that is analogous to the enthalpy under uniaxial stress, rather than internal energy, to model phase evolution under constant load, which is consistent with the load-controlled nature of the nanoindentor. Note that this free energy, which we call F, would correspond to the enthalpy if we considered the hydrostatic stress instead of uniaxial stress. We write this free energy (per unit $CaFe_2As_2$) as a function of the uniaxial stress and strain as well as the internal energy, obtained from DFT, as a function of the strain

$$F = \varphi_O E_O(\varepsilon_O) + \varphi_{cT} E_{cT}(\varepsilon_{cT}) - \sigma\varepsilon\Omega_0, \quad (5)$$

where φ_i is the phase fraction (i.e. the fraction of $CaFe_2As_2$ that is in phase "i"), E_i is the internal energy-per-unit phase (i.e. the energy per number of $CaFe_2As_2$), ε_i is the strain in the "i" phase and Ω_0 is the reference atomic volume of $CaFe_2As_2$. Here, we use the volume of the orthorhombic $CaFe_2As_2$ phase from our DFT simulations, which has lattice

constants a=5.60 $\AA$, b=5.49 $\AA$, and c=11.48 $\AA$, resulting in $\Omega_0 = 353\ \AA^3$. The energies of the two phases have been computed as a function of uniaxial strain as shown in Figure S8. Mechanical equilibrium of the two phases requires that the constraint:

$$\sigma = \frac{\partial e_O}{\partial \varepsilon_O} = \frac{\partial e_{cT}}{\partial \varepsilon_{cT}}, \tag{6}$$

is satisfied, where e_i is the strain energy per unit volume. Minimization of this free energy results in the appropriate volume fractions of the O and cT phases as well as the stress in the system under constant load. The strains in the individual phases can be determined using this phase fraction as:

$$\varepsilon = \varphi_O \varepsilon_O + \varphi_{cT} \varepsilon_{cT}. \tag{7}$$

In this definition, the strain in the cT phase, then, is the change in length per unit length in the cT phase relative to the O phase. This makes the mathematical implemnation easiest since both phases have the same reference length.

The minimization of the total free energy requires smooth functions while the DFT data, as shown in Supplementary Figs. 4 and 5, are discrete. To remedy this, we use a spline interpolant of the data to make suitable for numerical optimization. This is done by first selecting a fixed stress value and an estimate of the volume fractions of the phases which can be used, in conjunction with the Supplementary Equations (7) and (8) the numerical values for Supplementary Equation (5) can be determined. The minimization itself was carried out using the golden ratio search to find the minimum of Supplementary Equation (5) with the bounds that the phase fractions between 0 and 1. The evolution of the two phase fractions as a function of the applied strain obtained from this minimization are shown in Supplementary Fig. 6 demonstrating a non-linear relationship between phase fractions and the applied strain. The evolution of the strains

are plotted in Supplementary Fig. 7 clearly showing that the strains add up to the total strain.

The full solution of these equations results in a stress-strain curve shown in Fig. 2(c) of the manuscript. It is important to note a couple of points. First, the analysis uses DFT values of the O and cT phases at zero Kelvin, and has not been corrected for higher temperatures observed in the experiments. In addition, the DFT calculations have a stable cT structure at 0K, however in the experiments the cT structure spontaneously transforms back to the O phase as stress is released as the cT phase is not meta-stable at room temperature. Our composite model predicts the same thing because it assumes that the transition occurs to minimize the total free energy and ignores energy barriers. We expect some differences between theoretical models and experiments at lower temperatures.

Supplementary Note 4: Actuation work per-unit-volume

Even though the actuation testing has not been performed, it is still possible to calculate the actuation work per-unit-volume from a stress-strain curve as suggested in Lai, A., et al. *Science* **27,** 1505-1508 (2013)[4], and Huber, J. E., et al. *Proc. R. Soc. London Ser. A* **453,** 2185–2205 (1997)[5] (Note that Fig. 3 came from these two references.). They calculated the actuation work of shape memory alloys and ceramics simply by (transformation stress) × (transformation strain) because the actuation work per-unit-volume can be defined as the rectangular area below the stress-strain curve within the range of transformation strain. However, in the case of $CaFe_2As_2$, because the transformation stress increases in stage II, we can estimate the actuation work per-unit-volume as the area of trapezoid. This can be simply done by (average transformation stress) × (transformation strain). Here, the average transformation stress (σ_{PT}^{ave}) is the average of onset (σ_{PT}^{onset}) and offset (σ_{PT}^{offset}) transformation stress.

The onset and the offset of transformation can be defined by using the line-intercept methods. Each blue line in Supplementary Fig. 8(a) can be obtained by taking the linear regression on carefully selected linear-like region. Once we get the onset and offset stress/strain, we can approximate the area of stage II by (average transformation stress) × (transformation strain). The data points in Fig. 3 were calculated by this method from four experimental stress-strain curves and one DFT stress-strain curve in Supplementary Fig. 8(b). The calculated the actuation work per-unit-volume data are available in Supplementary Table 1.

Supplementary Note 5: Preliminary results on $LaRu_2P_2$

We recently tested $LaRu_2P_2$, which is another $ThCr_2Si_2$-type intermetallic compound (Supplementary Fig. 9(a)). This material also undergoes collapsed tetragonal phase transformation, and exhibit superelasticity. One of the notable mechanical properties is the ultra-high yield strength (~7 GPa) (Supplementary Fig. 9(b)). Thus, regardless of superelastic strain smaller than that of $CaFe_2As_2$, the actuation work is still excellent due to the ultra-high yield strength. Note that there is a region of decreasing contact stiffness (Supplementary Fig. 9(c)). Note that the decrease in contact stiffness is an another evidence of structural collapse because collapsed tetragonal phase is much stiffer than tetragonal phase. Our preliminary results show that superelasticity associated with collapsed tetragonal phase transition found in $CaFe_2As_2$ is only one manifestation of a wider class of such transitions found in significant number of $ThCr_2Si_2$-type intermetallic compounds. This is very important to search for industrial applicable (non-toxic) superelastic intermetallic compounds.

Supplementary References

1. Lee, S.-W., Han, S.-M. & Nix, W. D. Uniaxial compression of fcc Au nanopillars on an MgO substrate: The effects of prestraining and annealing. *Acta Mater*. **57,** 4404-4415 (2009).

2. Oliver, W. C. & Pharr, G. M. An improved technique for determining hardness and elastic modulus using load and displacement sensing indentation experiments. *J. Mater. Res*. **7,** 1564-1583 (1992).

3. Sypek, J. T., Weinberger, C. R., Vijayan, S., Aindow, M., Bud'ko, S. L., Canfield P. C. & Lee S.-W., Superelastic and micacecous deformation in the intermetallic compound $CaFe_2As_2$. *Scripta Mater*. **141**, 10-14 (2017).

4. Lai, A., Du, Z., Gan, C. L. & Schuh, C. A. Shape memory and superelastic ceramics at small scales. *Science* **27,** 1505-1508 (2013).

5. Huber, J. E., Fleck, N. A. & Ashby, M. F. The selection of mechanical actuators based on performance indices. *Proc. R. Soc. London Ser. A* **453,** 2185–2205 (1997).
