# Supplementary material for: Superelasticity and cryogenic linear shape memory effects of CaFe2As2
Source: Nat Commun. 2017 Oct 20;8:1083. doi: 10.1038/s41467-017-01275-z (PMC5715139; doi:10.1038/s41467-017-01275-z)
Supplement: Supplementary file 2 — Description of Additional Supplementary Files [file 41467_2017_1275_MOESM2_ESM.pdf]

Description of Additional Supplementary Files

File Name: Supplementary Movie 1
Description: Cyclic compression test with 20 cycles.
